# Prognostic Impact of Preoperative Naples Prognostic Score in Gastric Cancer Patients Undergoing Surgery

**DOI:** 10.3389/fsurg.2021.617744

**Published:** 2021-05-21

**Authors:** Jianping Xiong, Haitao Hu, Wenzhe Kang, Hao Liu, Fuhai Ma, Shuai Ma, Yang Li, Peng Jin, Yantao Tian

**Affiliations:** Department of Pancreatic and Gastric Surgery, National Cancer Center/National Clinical Research Center for Cancer/Cancer Hospital, Chinese Academy of Medical Sciences and Peking Union Medical College, Beijing, China

**Keywords:** gastric cancer, naples prognostic score, time-dependent ROC, prognosis, prognostic factors

## Abstract

**Background:** The Naples prognostic score (NPS) is established according to nutritional or inflammatory state, which has been identified as a new prognostic score for various malignant tumors. However, its prognosis prediction effect on gastric cancer (GC) patients is still unknown so far. The present work aimed to examine the NPS function in the prediction of GC prognosis.

**Methods:** In this study, patients undergoing surgery with no preoperative therapy were retrospectively examined from June 2011 to August 2019. Typically, the total cholesterol level, serum albumin content, neutrophil-to-lymphocyte ratio and lymphocyte-to-monocyte ratio were determined to calculate the NPS. Besides, the prognostic value of NPS was evaluated by survival analyses. Time-dependent receiver operating characteristic (t-ROC) curve analysis was also carried out to compare the prognostic value of the scoring systems.

**Results:** Altogether 1,283 cases were enrolled into the present work. NPS was markedly related to age, gender, tumor size, body mass index, vascular invasion, perineural invasion, and pTNM stage. Upon multivariate analysis, NPS was identified as an independent prognostic factor for the prediction of overall survival (OS) (*P* < 0.001). In subgroup analyses stratified by adjuvant chemotherapy or surgery alone, NPS was still the independent prognostic factor for OS in both groups (both *P* < 0.001). Furthermore, NPS exhibited higher accuracy in the prediction of OS than additional prognostic factors, as revealed by the results of t-ROC curve analysis.

**Conclusions:** NPS is a simple and useful scoring system that can be used to independently predict the survival of GC cases undergoing surgery.

## Introduction

Gastric cancer (GC) ranks the 5th place in terms of its morbidity, which affects 1,033,701 people annually; meanwhile, it also ranks the 3rd place among the causes of cancer-related deaths, with around 782,685 GC-related death cases being reported annually (1). Adjuvant therapy and surgical techniques have been greatly developed, but advanced GC patients still have dismal prognosis. As a result, it is of vital importance to develop a new preoperative prognostic marker to help to identify the surgery beneficial patients. Since Virchow first systematically reported the relationship of inflammation with cancer in the 19th century, more and more studies suggest the vital role of systemic inflammation in the tumor microenvironment (TME) (2, 3). Moreover, increasing studies indicate that inflammation in TME results in the proliferation, metastasis and angiogenesis of tumor cells, as well as antitumor immunity impairment and resistance to antitumor treatment (4). Accumulating studies report that, the inflammation-related prognostic scoring systems, including neutrophil-to-lymphocyte ratio (NLR), platelet-to-lymphocyte ratio (PLR), and lymphocyte-to-monocyte ratio (LMR), are related to the prognosis of cancer including GC, hepatocellular carcinoma (HCC) and esophageal squamous cell carcinoma (ESCC) (5, 6). However, the host condition also affects the prognostic ability of a single inflammation-related marker, and a single marker may even be misleading when the cutoff value is arbitrarily determined. Additionally, simple scoring systems that indicate the nutritional or immunological status before surgery, such as the prognostic nutritional index (PNI), the systemic inflammation score (SIS), and the controlling nutritional status (CONUT), have also been extensively employed in predicting prognosis (7, 8). Recently, an increasing number of studies report that NPS, which is established based on the preoperative total cholesterol (TC) content, serum albumin (Alb) content, LMR and NLR, represents a new inflammation-related prognostic scoring system. Researchers have demonstrated that NPS shows prognostic value for pancreatic cancer, colorectal cancer (CRC), lung cancer and osteosarcoma (9–11). Besides, NPS is more accurate than other prognostic factors in predicting survival (9, 10, 12). It takes into account the effects of systemic inflammation and nutritional status on cancer prognosis. As a result, NPS outperforms other single inflammatory or nutritional markers. Nonetheless, there is little research on the role of NPS in predicting the prognosis of GC patients.

As a result, the present retrospective cohort study was carried out aiming to determine the prognostic value of NPS among the GC patients and to investigate the relationships between NPS and additional clinicopathological characteristics.

## Patients and Methods

### Study Population

This retrospective study assessed the patients undergoing radical surgery due to GC from June 2011 to August 2019 at the Department of Pancreatic and Gastric Surgery, the National Cancer Center/Cancer Hospital, Chinese Academy of Medical Sciences and Peking Union Medical College. The patient inclusion criteria were as follows, those histologically or cytologically diagnosed with GC, those who were followed up for over 12 months, those with no inflammatory disorder or infection (normal white blood cell count, with no obvious symptom or sign of infectious disease), and those with no malignant tumor at other site or multiple primary malignant tumors. Moreover, the demographic, histopathological and laboratory variables of all patients were retrospectively analyzed, and relevant data were extracted from the database and patient records at our hospital.

Routine blood test was performed at a week before surgery. The results of blood tests conducted at a week before surgery were acquired from the Laboratory Database of National Cancer Center (Beijing, China). The preoperative information was extracted from each patient, which included gender, age, body mass index (BMI), TC content, serum Alb content, tumor size, absolute monocyte count (AMC), absolute neutrophil count (ANC) and absolute lymphocyte count (ALC). According to galizia et al.'s method, the serum Alb content, TC content, NLR and LMR were determined to calculate NPS (10) (Supplementary Figure 1). For patients with serum Alb content < 40 g/L, TC content ≤ 180 mg/dL, NLR > 2.96 and LMR < 4.44, the scores were 1; while for those with serum Alb content ≥ 40 g/l, TC content > 180 mg/dL, NLR ≤ 2.96 and LMR ≥ 4.44, the scores were 0. NPS represented the total score obtained from all the scores mentioned above. All cases were classified as 3 groups according to the NPS value, including group 0 (NPS, 0), group 1 (NPS, 1 or 2) and group 2 (NPS, 3 or 4). And subgroup analysis was stratified by adjuvant chemotherapy or surgery alone.

In this study, overall survival (OS), which was defined as the time between surgery and all-cause death or the last follow-up. Deaths due to causes other than GC or survivals till the end of observation period (last alive contact date) were considered as the censored observations of OS. The last follow-up was assessed in March 2020. Survival data were extracted from medical records or through telephone interviews during follow-up visits.

### Statistical Methods

Chi-square test was used to analyze categorical variables and *t*-tests were applied in analyzing the continuous variables. Survival curves were plotted by the Kaplan-Meier (KM) method, and log-rank test was utilized to analyze the differences. The significant variables identified from univariate analysis were incorporated in the multivariate Cox regression analysis. Concordance indices (C-indices) were calculated to evaluate the discriminatory power of the inflammation-based scores. And the time-dependent receiver operating characteristic (t-ROC) curves and the predicted values of area under the curve (AUC) were used to compare the prognostic value of NPS, SIS, CONUT and PNI (13). In addition to visually comparing the ROC curves, the AUC can be calculated. Sequential AUCs were compared between two scores using independent and identically distributed representations of AUC estimators. Each test was two-sided, and a difference of P <0.05 indicated statistical significance. The SPSS 18.0 (SPSS Inc., Chicago, IL, USA) and R ver. 4.0.2 (R Foundation for Statistical Computing, Vienna, Austria) softwares were employed for statistical analysis. Additionally, the R package “rms” was utilized to calculate the C index and the R package “timeROC” was adopted for t-ROC curve analysis. The present work gained approval from the Ethics Review Committee of National Cancer Center/Cancer Hospital, Chinese Academy of Medical Sciences and Peking Union Medical College and all patients signed informed consents.

## Results

### Patient Characteristics

In total, 1,283 GC cases were enrolled into the present work (Supplementary Figure 2), including 1,023 (79.7%) males and 260 (20.3%) females. The average age at the time of surgery of these patients was 61.1 (range, 23.0– 87.3) years. According to the pTNM staging system, 326 (25.5%) patients were at stage I, 322 (25.1%) at stage II and 635 (49.4%) at stage III, respectively. Seven hundred and twenty-two (60.2%) of these 1,283 cases received adjuvant chemotherapy. According to the NPS system, 256 cases had 0 point (ratio, 19.9%), 414 had 1 point (ratio, 32.3%), 340 had 2 points (ratio, 26.5%), 183 had 3 points (ratio, 14.3%), while 90 had 4 points (ratio, 7.0%), separately. As a result, 256 patients (59.8%) were assigned into group 0 (NPS 0), 273 (60.1%) in group 1 (NPS 1 or 2), and 273 (65.1%) in group 2 (NPS 3 or 4), respectively.

### Relationships of the Preoperative NPS System With Clinicopathological Characteristics

Table 1 summarizes the relationships of NPS with clinicopathological characteristics. NPS showed significant correlation with some clinicopathological characteristics. Additionally, a great NPS was related to the male sex, elder age (≥65.0 years), as well as reduced BMI (<18.5 kg/m2). With regard to tumor factors, NPS showed significant relationship with tumor size, vascular invasion, perineural invasion; however, there were no significant differences in lymphatic invasion, tumor differentiation, lauren classification, tumor location or adjuvant chemotherapy among these three NPS groups. In addition, NPS remarkably increased in patients with the serum Alb content (mg/dL) < 40 (*P* < 0.001), TC content (mg/dL) ≤ 180 (*P* < 0.001), LMR ≤ 4.44 (*P* < 0.001) and NLR > 2.96 (*P* < 0.001).

**Table 1:** Association of NPS and clinicopathological characteristics in patients with GC.

**Clinicopathological features**	**All cases (*n* = 1,283)**	**Group 0 (*n* = 256)**	**Group 1 (*n* = 754)**	**Group 2 (*n* = 273)**	***P*-value**
Age					<0.001
<65.0	69 (5.5)	175 (68.3)	478 (63.3)	136 (49.8)	
≥65.0	1,214 (94.5)	81 (32.7)	276 (36.7)	137 (50.2)	
Gender					<0.001
Male	1,023 (79.7)	174 (67.5)	597 (79.2)	252 (92.3)	
Female	260 (20.3)	82 (32.5)	157 (20.8)	21 (7.7)	
BMI (kg/m2)					<0.001
≥18.5	1,214 (94.5)	243 (30.9)	745 (98.8)	226 (82.9)	
<18.5	69 (5.5)	13 (69.1)	9 (1.2)	47 (17.1)	
Tumor size (cm)					<0.001
<3.0	267 (20.8)	71 (27.7)	161 (21.3)	35 (12.9)	
≥3.0	1,016 (78.2)	185 (72.3)	593 (79.5)	238 (87.1)	
Tumor differentiation					0.104
Differentiated	369 (28.8)	79 (30.9)	223 (29.5)	67 (22.4)	
Undifferentiated	914 (71.2)	177 (69.1)	531 (70.5)	206 (75.6)	
Lauren Classification					0.792
Intestinal-type	479 (37.3)	90 (35.2)	294 (39.1)	93 (34.1)	
Diffused-type	465 (36.3)	92 (36.0)	276 (36.6)	95 (35.0)	
Mixed	339 (26.4)	74 (28.8)	184 (24.3)	85 (30.9)	
Lymphatic invasion					0.068
Negative	636 (49.6)	137 (53.4)	379 (50.3)	120 (44.1)	
Positive	647 (50.4)	119 (46.6)	375 (49.7)	153 (55.9)	
Vascular invasion					0.001
Negative	778 (60.7)	171 (66.8)	462 (62.2)	145 (53.2)	
Positive	505 (39.3)	85 (33.2)	292 (38.7)	128 (46.8)	
Perineural invasion					0.004
Negative	628 (49.0)	141 (55.1)	371 (49.3)	116 (42.5)	
Positive	655 (51.0)	115 (44.9)	383 (50.7)	157 (57.5)	
Tumor location					0.082
Upper	48 (28.6)	58 (22.7)	30 (27.8)	80 (29.5)	
Middle /Lower	183 (71.4)	198 (77.3)	120 (72.2)	193 (70.5)	
pTNM stage					<0.001
I	326 (25.5)	89 (34.7)	183 (24.4)	54 (19.8)	
II	322 (25.1)	53 (20.7)	214 (28.3)	55 (20.1)	
III	635 (49.4)	114 (44.6)	357 (47.3)	164 (60.1)	
Adjuvant chemotherapy					0.387
No	511 (39.8)	106 (41.4)	302 (40.1)	103 (37.7)	
Yes	772 (60.2)	150 (58.6)	452 (59.9)	170 (62.3)	
Serum albumin (mg/dL)					<0.001
≥ 40	890 (69.4)	256 (100.0)	577 (76.5)	57 (20.9)	
<40	393 (30.6)	0 (0)	177 (23.5)	216 (79.1)	
Total cholesterol (mg/dL)					<0.001
>180	515 (40.2)	256 (100.0)	222 (29.5)	37 (13.6)	
≤ 180	768 (59.8)	0 (0)	532 (70.5)	236 (86.4)	
Neutrophil: lymphocyte ratio					<0.001
≤ 2.96	1,029 (80.2)	256 (100.0)	686 (91.0)	87 (31.9)	
>2.96	254 (19.8)	0 (0)	68 (9.0)	186 (68.1)	
Lymphocyte: monocyte ratio					<0.001
>4.44	845 (65.9)	256 (100.0)	533 (70.7)	56 (20.5)	
≤ 4.44	438 (34.1)	0 (0)	221 (29.3)	217 (79.5)	

*GC, gastric cancer; NPS, naples prognostic score; BMI, body mass index*.

### OS Examined Based on NPS

**Figure 1:**
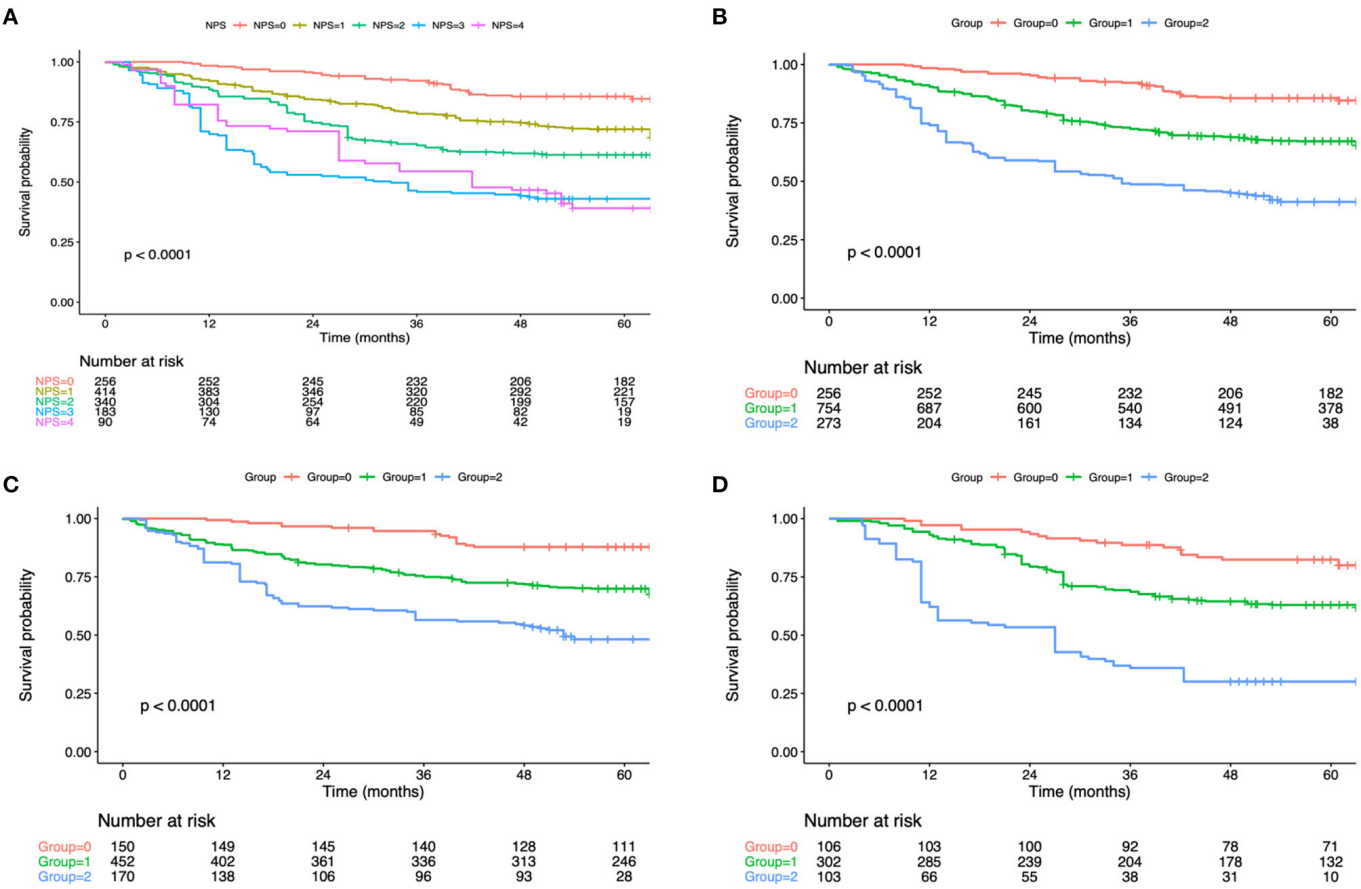
**(A)** Kaplan–Meier survival analyses of OS according to the NPS score. **(B)** Kaplan–Meier survival analyses of OS according to the NPS group. OS, overall survival; NPS, naples prognostic score. **(C)** Association of the NPS with the OS in the adjuvant chemotherapy group. **(D)** Association of the NPS with the OS in the surgery alone group. OS, overall survival; NPS, naples prognostic score.

**Figure 2:**
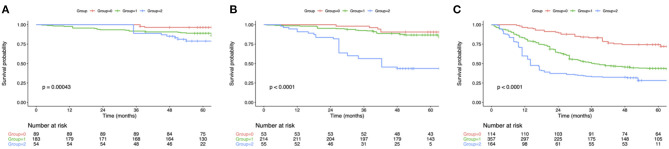
Kaplan-Meier analysis of OS of GC patients at each pTNM stage according to the NPS. **(A)** Association of the NPS with OS of patients with stage I GC. **(B)** Association of the NPS with OS of patients with stage II GC. **(C)** Association of the NPS with OS of patients with stage III GC. OS, overall survival; NPS, naples prognostic score; GC, gastric cancer.

The OS curve was statistically analyzed, as shown in Figure 1. For all the enrolled patients, their 1-, 3- and 5-year OS rates were 89.1, 70.6, and 46.7%, separately, and the median OS was 51.9 months. With regard to OS, the median OS was 67.7, 52.5 and 35.7 months for groups 0, 1 and 2, respectively. According to KM survival analysis, NPS score 0–4 was tightly related to OS, and the elevation of preoperative NPS by 1 point was markedly related to the poor OS (Log-rank *P* <0.001; statistic power >80%; Figure 1A). Also, KM survival analyses indicated that OS was markedly shortened with the elevation of NPS group in a step-wise manner (Log-rank *P* < 0.001; statistic power >80%; Figure 1B). Furthermore, significant difference was observed in OS based on NPS in single surgery group as well as postoperative adjuvant chemotherapy group (Figures 1C,D). When stratified by pTNM stage, the most significant differences in OS and RFS were observed in the stage III subgroup based on NPS system (Figure 2).

### Univariate and Multivariate Analyses on the Prognostic Predictors in GC Cases

**Figure 3:**
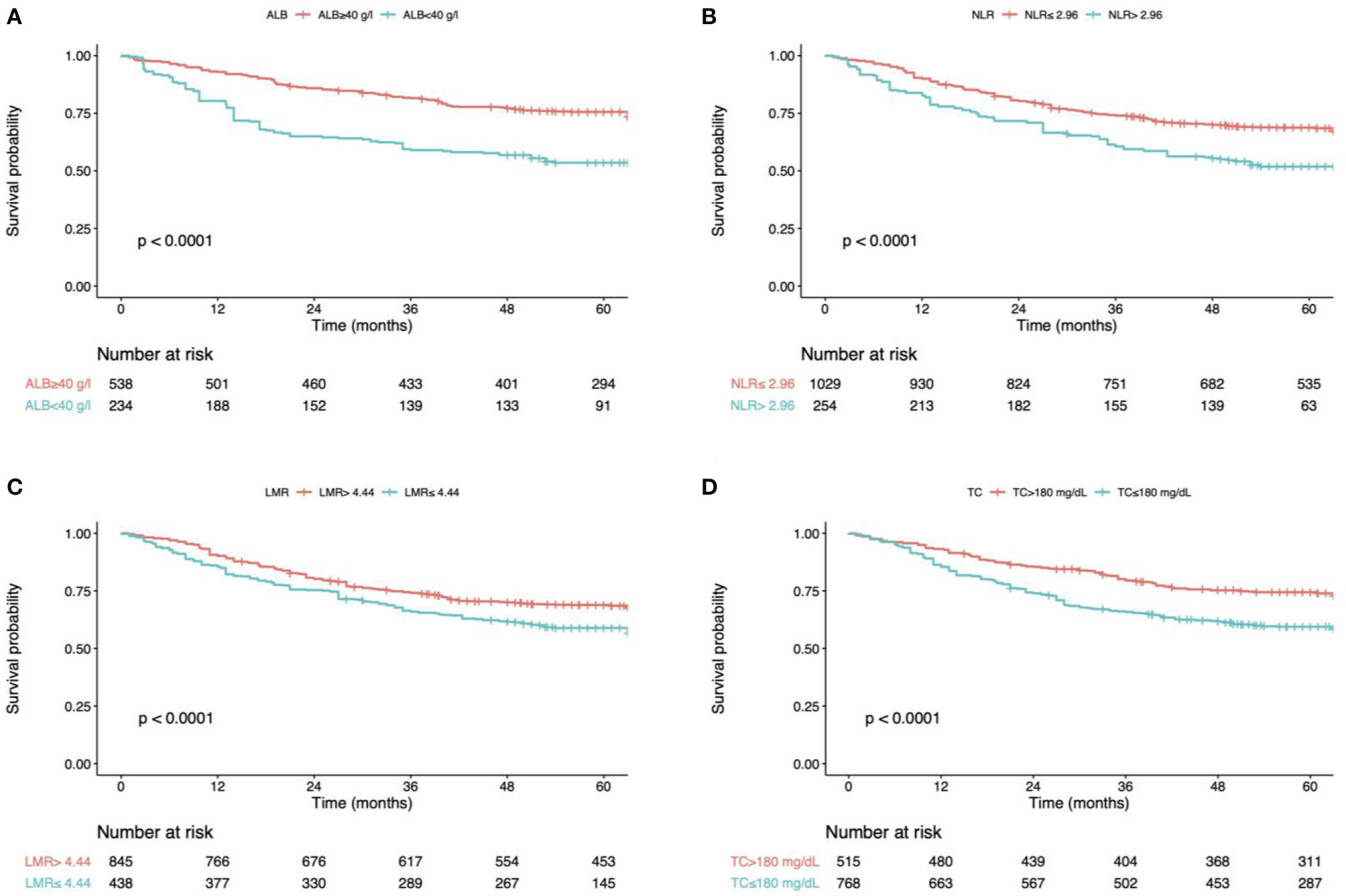
**(A)** overall survival curves according to the preoperative ALB. **(B)** overall survival curves according to the preoperative NLR. **(C)** overall survival curves according to the preoperative LMR. **(D)** overall survival curves according to the preoperative TC. ALB, albumin; TC, total cholesterol; NLR, neutrophil-to-lymphocyte ratio; LMR, lymphocyte-to-monocyte ratio.

In univariate analysis, patients in group 0 (low NPS) showed markedly extended OS compared with that in groups 1 and 2 (both *P* < 0.001, respectively; Table 2). As for patient characteristics, the elder age (≥ 65.0 years) was markedly associated with dismal OS (HR = 1.78, *P* < 0.001). Additionally, the decreased BMI (<18.5 kg/m2) was related to dismal OS (HR = 1.69, *P* < 0.001). In terms of the tumor characteristics, the undifferentiated (HR = 1.58, *P* < 0.001), diffused-type (HR = 1.23, *P* = 0.001), vascular invasion (HR = 1.70, *P* < 0.001), lymphatic invasion (HR = 1.93, *P* < 0.001) and perineural invasion (HR = 1.83, *P* < 0.001) showed marked correlation with dismal prognostic outcomes. NPS was identified as the independent factor to predict the OS upon multivariate analysis (Figure 1B). In addition, OS was markedly impaired among cases with serum Alb content (mg/dL) < 40 (HR = 0.72, *P* = 0.001), LMR ≤ 4.44 (HR = 1.71, *P* < 0.001) and NLR> 2.96 (HR = 1.59, *P* < 0.001) (Figure 3; Table 2). In addition, more independent factors for prognosis prediction included an old age, female sex, decreased BMI, pTNM stage, undifferentiated, diffused-type and the presence of vascular invasion, lymphatic invasion and perineural invasion (Table 2).

**Table 2:** Univariate and multivariate analysis of clinicopathologic variables in relation to OS in patients with GC.

**Clinicopathological features**	**Univariate analysis**	***P*-value**	**Multivariate analysis**	***P*-value**
**Age**				
<65.0	Reference		Reference	<0.001
≥65.0	1.78 (1.41, 2.25)	<0.001	1.60 (1.44, 1.79)	
**Gender**				
Male	Reference			
Female	0.85 (0.46, 1.62)	0.182		
**BMI (kg/m2)**				
≥18.5	Reference		Reference	
<18.5	1.69 (1.27, 2.13)	<0.001	1.54 (1.21, 1.80)	<0.001
**Tumor size (cm)**				
<3.0	Reference		Reference	
≥3.0	2.03 (1.50, 2.52)	<0.001	1.27 (0.76, 1.63)	0.438
**Tumor differentiation**				
Differentiated	Reference		Reference	
Undifferentiated	1.58 (1.32, 2.15)	<0.001	1.47 (1.13, 1.92)	0.008
**Lauren Classification**				
Intestinal-type	Reference		Reference	
Diffused-type	1.23 (1.05, 1.78)	0.001	1.16 (1.01, 1.45)	0.009
Mixed	1.04 (0.67, 1.51)	0.871		
**Lymphatic invasion**				
Negative	Reference		Reference	
Positive	1.93 (1.42, 2.88)	<0.001	1.60 (1.32, 2.12)	0.004
**Vascular invasion**				
Negative	Reference		Reference	
Positive	1.70 (1.28, 2.21)	<0.001	1.55 (1.32, 1.83)	<0.001
**Perineural invasion**				
Negative	Reference		Reference	
Positive	1.83 (1.49, 2.65)	<0.001	1.62 (1.43, 1.90)	0.011
**Tumor location**				
Upper	Reference			
Middle /Lower	0.85 (0.39, 1.61)	0.230		
**pTNM stage**				
I	Reference		Reference	
II	2.14 (1.45, 2.67)	0.001	1.83 (1.32, 2.28)	<0.001
III	5.47 (2.31, 8.41)	0.007	3.22 (2.10, 4.19)	<0.001
**Adjuvant chemotherapy**				
No	Reference			
Yes	0.79 (0.45, 1.48)	0.173		
**Serum albumin (mg/dL)**				
<40	Reference		Reference	
≥40	0.65 (0.52, 0.94)	0.001	0.72 (0.56, 0.90)	<0.001
**Total cholesterol (mg/dL)**				
>180	Reference		Reference	
≤ 180	1.52 (1.14, 2.10)	0.001	1.46 (1.10, 1.87)	<0.001
**Neutrophil: lymphocyte ratio**				
≤ 2.96	Reference		Reference	
>2.96	1.69 (1.28, 2.26)	0.003	1.59 (1.23, 1.92)	<0.001
**Lymphocyte: monocyte ratio**				
>4.44	Reference		Reference	
≤ 4.44	1.78 (1.33, 2.32)	<0.001	1.71 (1.22, 2.01)	<0.001
**NPS**				
0	Reference		Reference	
1	2.68 (1.45, 4.01)	0.002	2.21 (1.27, 3.31)	<0.001
2	3.82 (1.89, 6.80)	<0.001	3.45 (1.43, 5.17)	<0.001

*OS, overall survival; GC, gastric cancer; BMI, body mass index; NPS, naples prognostic score*.

### Prognostic Value of NPS

**Figure 4:**
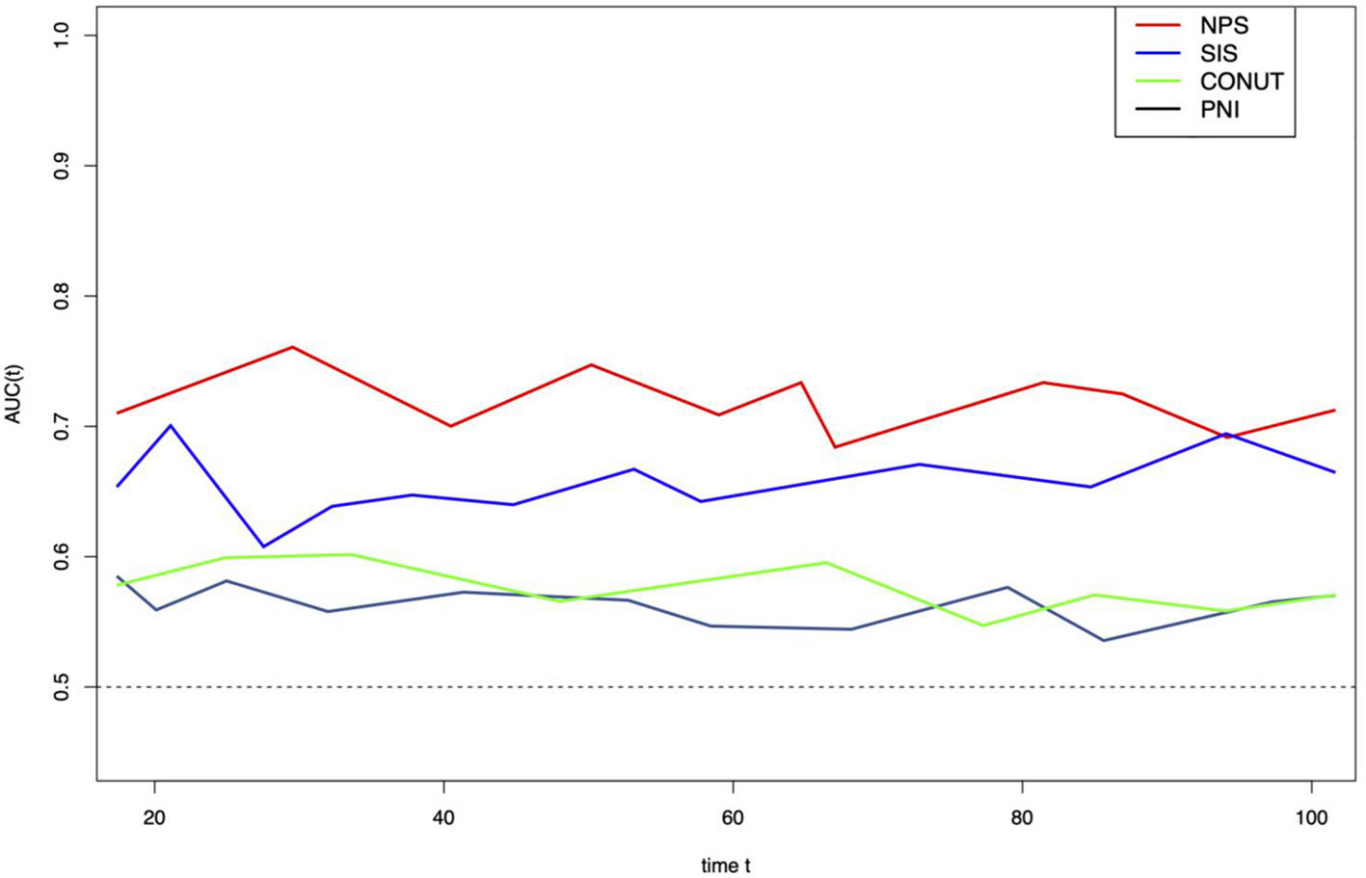
Time-dependent ROC curves of NPS, SIS, CONUT, and PNI for prediction of overall survival. The horizontal axis represents year after surgery, and the vertical axis represents the estimated AUC for survival at the time of interest. NPS, naples prognostic score; SIS, systemic inflammation score; CONUT, controlling nutritional status; PNI, prognostic nutritional index.

In this study, the prognostic value of NPS was compared with that of more prognostic factors (PNI, CONUT and SIS). As suggested by results of t-ROC curve analysis to predict OS by different scoring systems, and the AUC value was high for NPS compared with those for other scoring systems (Figure 4). Typically, the AUC values in the prediction of 5-year OS were 0.708, 0.549, 0.625, and 0.580 for NPS, PNI, SIS and CONUT, respectively (Figure 4).

## Discussion

As suggested by this work, the preoperative NPS was related to gender, age, BMI, tumor size, perineural invasion and vascular invasion in GC patients. NPS might serve as an independent factor to predict the OS for GC cases, and a large NPS was possibly related to the dismal prognosis. Additionally, NPS was more accurate than other prognostic scoring systems developed in previous studies (PNI, SIS and CONUT) in predicting OS.

The biological mechanism by which NPS affects patient prognosis is possibly dependent on the serum Alb content, serum TC content, neutrophil count, monocyte count and lymphocyte count, which are easily measured before surgery. Hypoalbuminemia suggests the low nutritional status along with the elevated inflammatory level, and it may negatively affect the surivval of GC patients. In addition, hypoalbuminemia can reduce the transport of substances like fatty acid and cholesterol as well as the scavenging of free oxygen radicals, and these show adverse effects on OS. The TC content is identified to be related to patient survival and tumor development in a variety of cancer types, such as GC, since the plasma TC content and calorie intake are reduced in tumor tissues0 (14). LMR is determined by lymphocytes and monocytes, whereas NLR is related to neutrophils and lymphocytes. Biologically, LMR and NLR were possibly revealed by the lymphocyte, monocyte and neutrophil functions. And lymphocytosis indicates the immunological status, and it enhances the anticancer response against the proliferation, new vessel formation and migration of cancer cells (15). In this paper, an example is the clinical significance of tumor-infiltrating lymphocytes (TILs), which are related to the superior prognosis for various cancer types, and this may be related to the anticancer effects induced by TILs and the suppression of angiogenesis (15, 16). Therefore, cancer patients with lymphopenia had dismal survival (17). Neutrophils can invade primary tumor, release the pro-angiogenic factors and promotes tumor cell migration along the new blood vessels, thus facilitating tumor metastasis. And neutrophils are reported to enhance the adhesion of circulating cancer cells with end organs, which thereby increases the risk of metastatic seeding. The circulating monocytes facilitate cancer growth and decrease the immune monitoring (18). Further, studies have shown that monocytes may promote tumor cell metastasis via the tumor-monocyte-endothelial interaction (19). Thus, monocytes and neutrophils enhanced the proliferation of cancer cells and regulated the TME, thus facilitating tumor metastasis, invasion and new vessel formation. Therefore, the high neutrophil count while low monocyte count in cancer patients indicate dismal OS.

As for the SIS scoring system that developed on the basis of preoperative LMR and Alb contents, it is identified to be related to survival of a variety of cancers and can serve as a creditable inflammatory-based scoring system (8, 20). It was discovered by Melling and colleague that, GPS might also independently predict the long-term prognosis for GC patients receiving surgery (21). CONUT is determined based on the total lymphocyte count, serum Alb content and TC content, is recognized as a useful approach to assess nutritional status (22). Moreover, it was discovered by Kuroda and colleagues that, CONUT was an efficient approach to estimate the the nutritional status and to predict the long-term OS for GC cases who underwent radical surgery (7). PNI, which predicts the nutritional and immune statuses, is a scoring system used to evaluate patient general condition and an efficient factor to predict the long-term survival for GC case (23). In addition, Galizia et al. revealed that NPS possibly outperformed the other scoring systems (like PNI, SIS and CONUT) in predicting the CRC survival (10). Nakagawa et al. indicated that NPS was more sensitive than CONUT in predicting the OS in pancreatic cancer (9). As found in this work, NPS outperformed PNI, SIS and CONUT in predicting the prognosis for GC cases who underwent radical surgery.

Compared with the existing tools to target immunonutritional interventions, our system is superior in that, by combining the oncological, nutritional, and immunological parameters, it outperforms the other existing nutritional indices in predicting the postoperative adverse events. And it targets the immunonutritional intervention to patients who may benefit the most. The results of our study indicated that early inflammation control and nutritional support might improve the prognosis for cancer patients. Preoperative identification of patient status could have several uses in clinical practice, including prognostic stratification and treatment. Early detection and improvement of malnutrition and inflammation may result in better patient outcomes (24).

Certain limitations should be noted in this study. Firstly, selection bias was inevitable due to the retrospective nature, even though the samples were strictly selected according to the inclusion and exclusion criteria. And the significance of NPS needs to be validated using other cohorts. Secondly, patients who received NACT were eliminated from this study, but it was difficult to guarantee the identical patient status prior to blood sample collection, and our findings did not apply to GC cases after NACT.

## Conclusions

To sum up, this work suggests that preoperative NPS can serve as a simple and useful predictor to predict the prognosis of GC. Besides, NPS is also utilized as one part of the preoperative prognosis stratification as well as postoperative follow-up for the development of individual treatment for GC.

## Data Availability Statement

The original contributions presented in the study are included in the article/Supplementary Material, further inquiries can be directed to the corresponding authors.

## Ethics Statement

The studies involving human participants were reviewed and approved by Ethics Review Committee of National Cancer Center/Cancer Hospital, Chinese Academy of Medical Sciences and Peking Union Medical College. The patients/participants provided their written informed consent to participate in this study.

## Author Contributions

JX conceived the study and wrote the manuscript. WK, HH, and FM searched the database, reviewed the studies, and collected the data. HL and SM performed the statistical analyses. YL and PJ performed revision of the manuscript. YT arranged for and provided the funding for this work and is the guarantor for this study. All authors contributed to the article and approved the submitted version.

## Conflict of Interest

The authors declare that the research was conducted in the absence of any commercial or financial relationships that could be construed as a potential conflict of interest.

## Supplementary Material

Supplementary Figure 1 Calculation of the Naples prognostic score.

Supplementary Figure 2 Study design. GC, gastric cancer.

## Abbreviations

GC: Gastric cancer
HCC: hepatocellular carcinoma
ESCC: esophageal squamous cell carcinoma
OS: overall survival
NPS: naples prognostic score
SIS: systemic inflammation score
CONUT: controlling nutritional status
PNI: prognostic nutritional index
ALB: albumin
TC: total cholesterol
NLR: neutrophil-to-lymphocyte ratio
PLR: platelet-to-lymphocyte ratio
LMR: lymphocyte-to-monocyte ratio
BMI: body mass index
AUC: area under the curve
ROC: receiver operating characteristic
GC: Gastric cancer
HCC: hepatocellular carcinoma
ESCC: esophageal squamous cell carcinoma.
