# Supplementary figures and images for: Prognostic Impact of Preoperative Naples Prognostic Score in Gastric Cancer Patients Undergoing Surgery
Source: Front Surg. 2021 May 21;8:617744. doi: 10.3389/fsurg.2021.617744 (PMC8176017; doi:10.3389/fsurg.2021.617744)

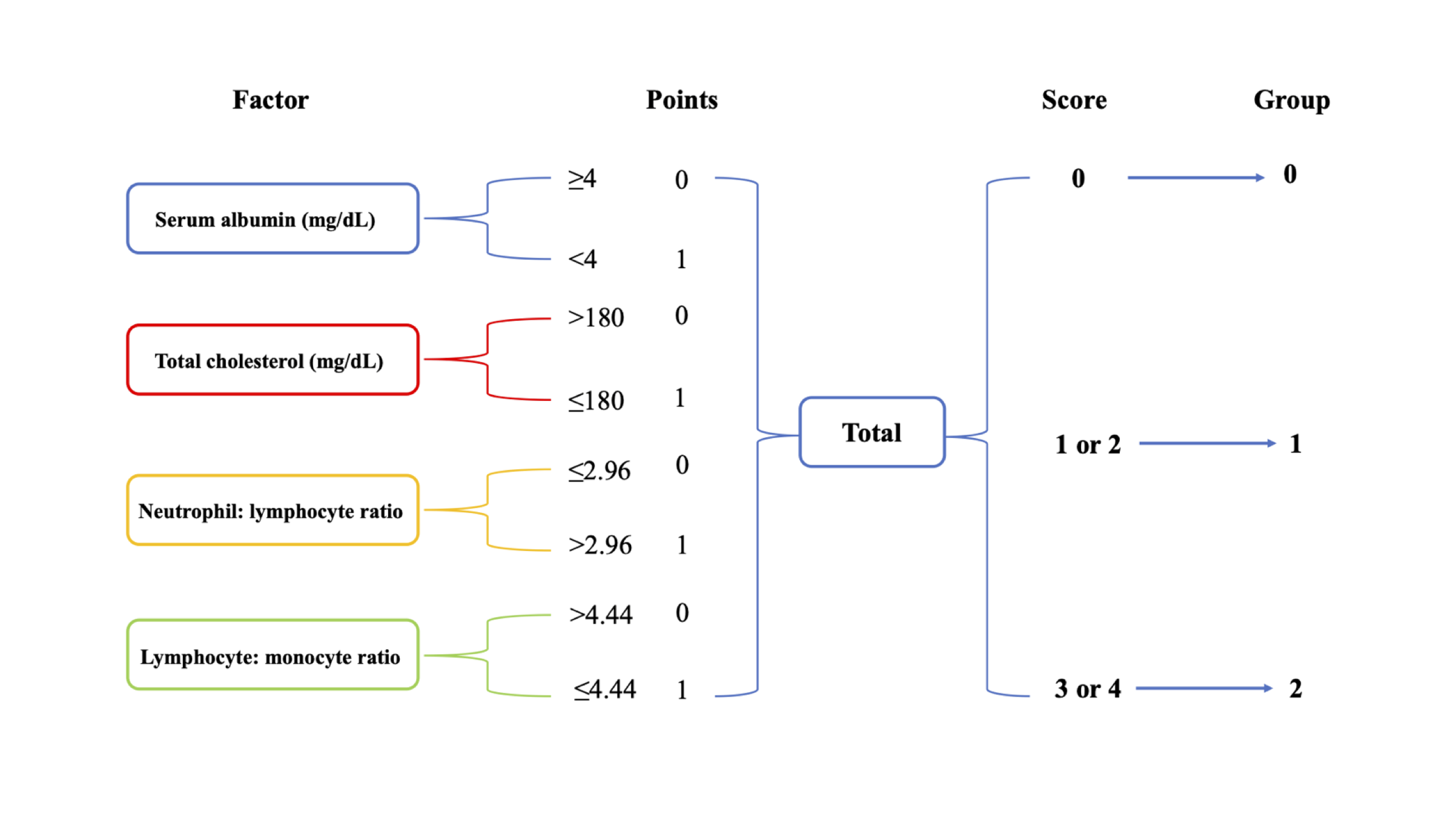

Supplement: Supplementary Figure 1 — Calculation of the Naples prognostic score. [file Image_1.TIFF]

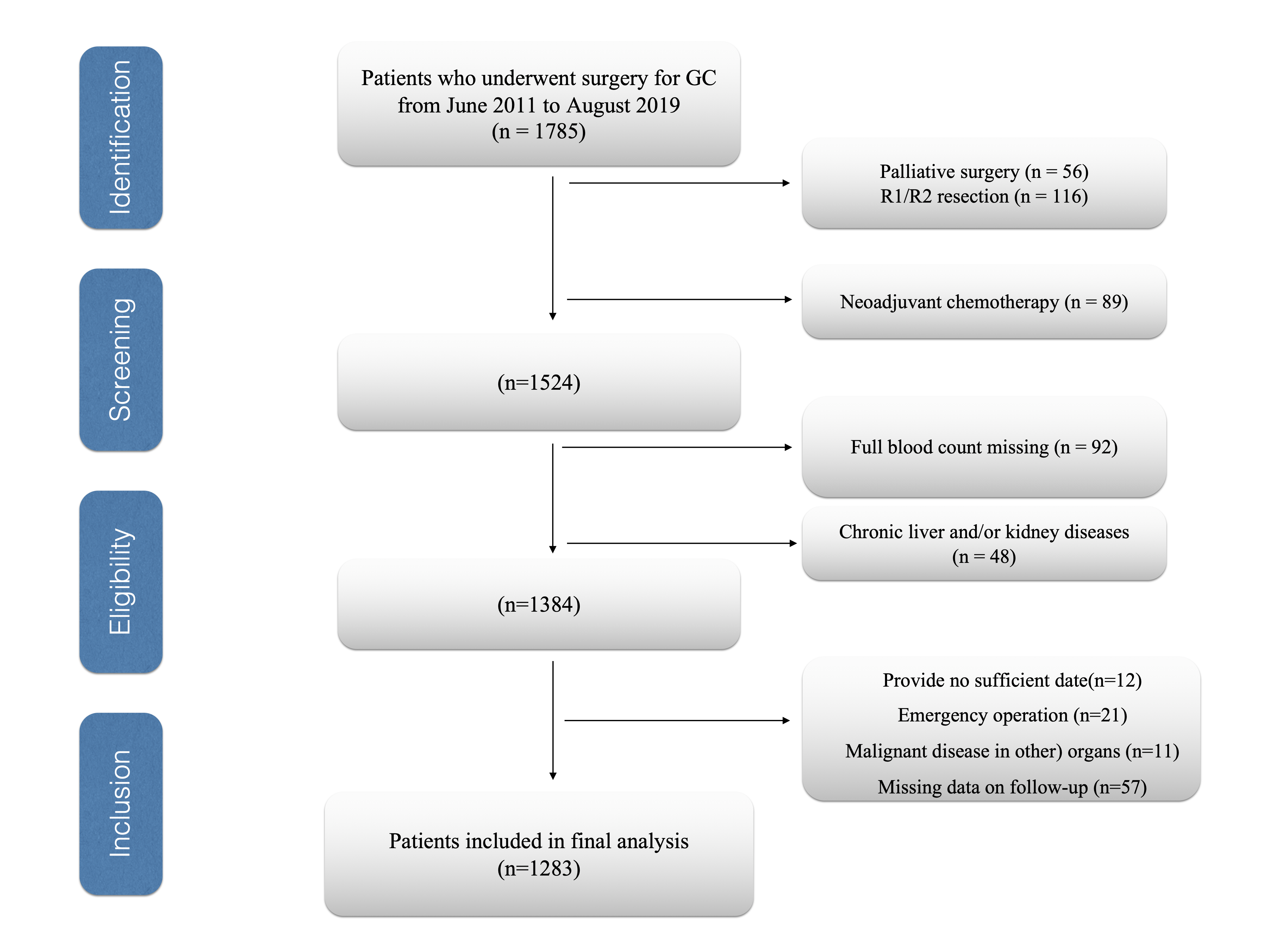

Supplement: Supplementary Figure 2 — Study design. GC, gastric cancer. [file Image_2.TIFF]
